# Comparison of Radiographic and Patient-Reported Outcomes in Robotic-Assisted Versus Manual Total Knee Arthroplasty Using Medial-Congruent Bearing

**DOI:** 10.3390/jcm15020806

**Published:** 2026-01-19

**Authors:** Wen-Chien Wang, Yu-Tsung Lin, Kun-Hui Chen, Cheng-Hung Lee, Cheng-Chi Wang, Chung-Yuh Tzeng, Kelly Vince

**Affiliations:** 1 Department of Orthopedics, Taichung Veterans General Hospital, Taichung 40705, Taiwan; wcwang0526@vghtc.gov.tw (W.-C.W.); dan90008@vghtc.gov.tw (Y.-T.L.); khc@vghtc.gov.tw (K.-H.C.); 298f@vghtc.gov.tw (C.-H.L.); 2 Department of Post-Baccalaureate Medicine, College of Medicine, National Chung Hsing University, Taichung 402202, Taiwan; 3 Department of Food Science and Technology, Hung Kuang University, Taichung 43302, Taiwan; 4 Department of Rehabilitation, Jen-Teh Junior College of Medicine, Nursing, and Management, Miaoli 356606, Taiwan; 5 Institute of Biomedical Sciences, National Chung Hsing University, Taichung 402202, Taiwan; 6 Whangarei Hospital, Whangarei 0110, New Zealand

**Keywords:** robotic-assisted total knee arthroplasty, manual total knee arthroplasty, medial-congruent bearing, patient-reported outcome, posterior tibial slope

## Abstract

**Background**: Total knee arthroplasty (TKA) effectively relieves pain in end-stage osteoarthritis, yet a proportion of patients remain dissatisfied despite advances in surgical technique. Medial-congruent (MC) bearings are designed to recreate native medial-pivot kinematics, which depend on appropriate medial compartment soft tissue tension. Robotic-assisted TKA (RA-TKA) has been shown to improve the accuracy and soft tissue balance. However, evidence of its additional benefits in MC TKA remains limited. **Methods**: We retrospectively identified consecutive primary TKAs with the same MC bearing performed between April 2022 and June 2024 at a tertiary center. After performing 1:1 propensity score matching to reduce baseline imbalance, 36 patients who received RA-TKA and 36 who underwent manual TKA (M-TKA) were included. Primary outcomes were evaluated with the 12-month Oxford Knee Score (OKS) and KOOS-JR. Secondary outcomes included radiographic alignment parameters, outlier rates, operative time, liner thickness, and hospital stay. **Results**: Baseline characteristics and liner thickness were comparable, and operative time was longer in the RA-TKA group than in the M-TKA group. Both RA-TKA and M-TKA produced significant 12-month improvements in OKS and KOOS-JR with no difference in mean scores. RA-TKA had fewer posterior tibial slope outliers (mean slope 4.3° ± 1.8 vs. 5.9° ± 3.1; outlier rate 16.7% vs. 41.7%; *p* = 0.02), whereas coronal alignment parameters did not differ between groups. **Conclusions**: RA-TKA with MC bearing provides functional outcomes comparable to M-TKA and may decrease sagittal alignment variability; long-term follow-up studies are needed to determine whether this potential benefit translates into sustained functional gains or improved implant survivorship.

## 1. Introduction

Total knee arthroplasty (TKA) is an effective treatment for end-stage knee osteoarthritis with functional disability. Nevertheless, despite advances in surgical technique and implant design, a certain proportion of patients still report dissatisfaction after TKA [1,2,3], underscoring a persistent gap between technical success and patient experience. This gap has focused attention on several modifiable, surgeon-controlled domains: adequate soft tissue balance, implant selection, and the accuracy of component positioning.

Among contemporary options, medial stabilized design, including medial-congruent (MC) or medial-pivot (MP) bearing, have gained popularity in recent years [4]. This construct features a highly conforming medial compartment in the sagittal plane, creating a “ball-and-socket” articulation that promotes medial-pivot motion. Comparative studies and meta-analyses suggest that medial-stabilized bearing designs yield outcomes comparable to, and in some series better than, traditional posterior-stabilized implants, although findings are not uniform across cohorts and methods [5,6,7,8].

Appropriate medial compartment tension is critical to reproducing the intended pivoting kinematics. Excessive medial flexion gap (medial laxity) after medial-congruent TKA has been linked to subjective instability and worse patient-reported outcomes, highlighting the importance of precise bone cuts and adequate soft tissue tension [9].

In parallel, RA-TKA has been adopted to improve the accuracy and reproducibility of bone resections and consistency of soft tissue tension [10]. Multiple comparative studies and meta-analyses report that robotic-assisted TKA (RA-TKA) improves mechanical alignment and component positioning and reduces radiographic outliers [11,12,13].

Whether these radiographic gains translate into clinically important benefits remains debated. Some cohorts have observed better patient-reported outcomes with RA-TKA [13], whereas others report similar scores to conventional instrumentation at mid-to-long-term follow-up [12,14,15]. A 12-month randomized controlled trial using the same implant design reported broadly similar patient-reported outcomes between robotic-assisted and manual TKA, although pain improvement differed between groups [16]. Potential factors contributing to the variability among these study results include alignment philosophy, implant family, and perioperative care. Moreover, much of the literature centers on cruciate-retaining or posterior-stabilized constructs, with comparatively limited data on the interplay between robotics and MC design when both are applied using a consistent surgical approach and standardized pathways.

Therefore, we hypothesized that, compared with conventional manual total knee arthroplasty (M-TKA), RA-TKA using an MC bearing would produce superior outcomes. By isolating implant choice and standardizing perioperative care, this study aimed to determine whether robotic assistance confers measurable radiographic and clinical advantages.

## 2. Materials and Methods

This retrospective cohort study analyzed prospectively collected data from a single tertiary medical center and was approved by the Institutional Ethics Review Committee (Ref. No. CE24354A). The data were accessed for research purposes in March 2025 and can be accessed during or after data collection. The need for consent was waived by the ethics committee. All patients had a minimum follow-up of 12 months. The primary outcomes were the 12-month Oxford Knee Score (OKS) and KOOS-JR; secondary outcomes included coronal component alignment, posterior tibial slope, outlier rates, operative time, and length of stay.

We reviewed consecutive primary total knee arthroplasties (TKAs) performed with the same medial-congruent implant (Persona^®^ MC, Zimmer Biomet, Warsaw, IN, USA) between April 2022 and June 2024. The robotic-arm system (ROSA Knee, Zimmer Biomet) was introduced in July 2022 in our institute. Allocation to robotic-assisted versus conventional TKA occurred through surgeon–patient shared decision-making. To mitigate learning-curve effects, the first 10 ROSA cases for each surgeon were excluded from enrollment. Inclusion criteria were Kellgren and Lawrence grade III and IV with neutral to varus limb alignment (preoperative hip–knee–ankle [HKA] angle ≥ −3°, positive values indicating varus). Exclusion criteria were prior ipsilateral knee arthroplasty or high tibial osteotomy, inflammatory arthropathy, valgus deformity (HKA < −3°), and bone defects requiring augments or stems. Simultaneous bilateral TKAs were also excluded. To reduce baseline imbalance between groups, 1:1 propensity score matching (PSM) was performed using age, sex, and body mass index (BMI) as covariates.

### 2.1. Surgical Techniques

Restricted kinematic alignment (rKA) targeting a hip–knee–ankle (HKA) angle within ±3° of neutral was applied in both the M-TKA and RA-TKA groups. All procedures were performed by four experienced arthroplasty surgeons with 11, 15, 26, and 28 years of independent practice, respectively. A medial parapatellar or midvastus approach was used according to the surgeon’s preference. The anterior cruciate ligament was resected in all cases; management of the posterior cruciate ligament was at the surgeon’s discretion based on intraoperative flexion–extension balance after bone resections.

In the manual group, resections were planned to reproduce each patient’s pre-arthritic joint line while constraining the overall limb alignment within safe limits (target hip–knee–ankle angle ±3°; joint-line obliquity within ±5°). Preoperative long-leg radiographs were used to estimate the lateral distal femoral angle (LDFA) and medial proximal tibial angle (MPTA). Distal femoral and proximal tibial cuts were performed using the measured-resection technique with caliper verification, compensating for cartilage wear and saw-blade thickness. The tibial cut was limited to ≤5° varus relative to the mechanical axis. Femoral component rotation was set at 3° external rotation relative to the posterior condylar axis [17].

In the robotic-assisted group, a CT-free, semi-active robotic-arm system (ROSA^®^ Knee, Zimmer Biomet) was used. Preoperative full-length standing radiographs were uploaded to generate the surgical plan, including predicted component sizes and resection levels. Intraoperatively, optical trackers were affixed, and anatomic landmarks were registered to calibrate the system, which then guided bone resections. The same restricted kinematic alignment strategy and boundaries were applied through robotic planning and intraoperative feedback to achieve equivalent gap balancing and component positioning.

All cases in both groups underwent patella resurfacing and cemented implantation. Procedures were performed under spinal anesthesia or general anesthesia. Venous thromboembolism prophylaxis consisted of aspirin 100 mg once daily for 14 days per institutional protocol. Other perioperative analgesia and postoperative care followed standardized institutional pathways.

Demographic and perioperative variables (age, sex, body mass index [BMI], polyethylene insert thickness, operative time, and length of stay) were extracted from electronic medical records. Functional outcomes were obtained via telephone interviews preoperatively and at 12 months postoperatively using the Knee Injury and Osteoarthritis Outcome Score for Joint Replacement (KOOS-JR; 0–100, higher is better) and the Oxford Knee Score (OKS; 0–48, higher is better). The minimum clinically important differences (MCIDs) for KOOS-JR and OKS were 16 and 5 points, respectively [18,19].

**Figure 1:**
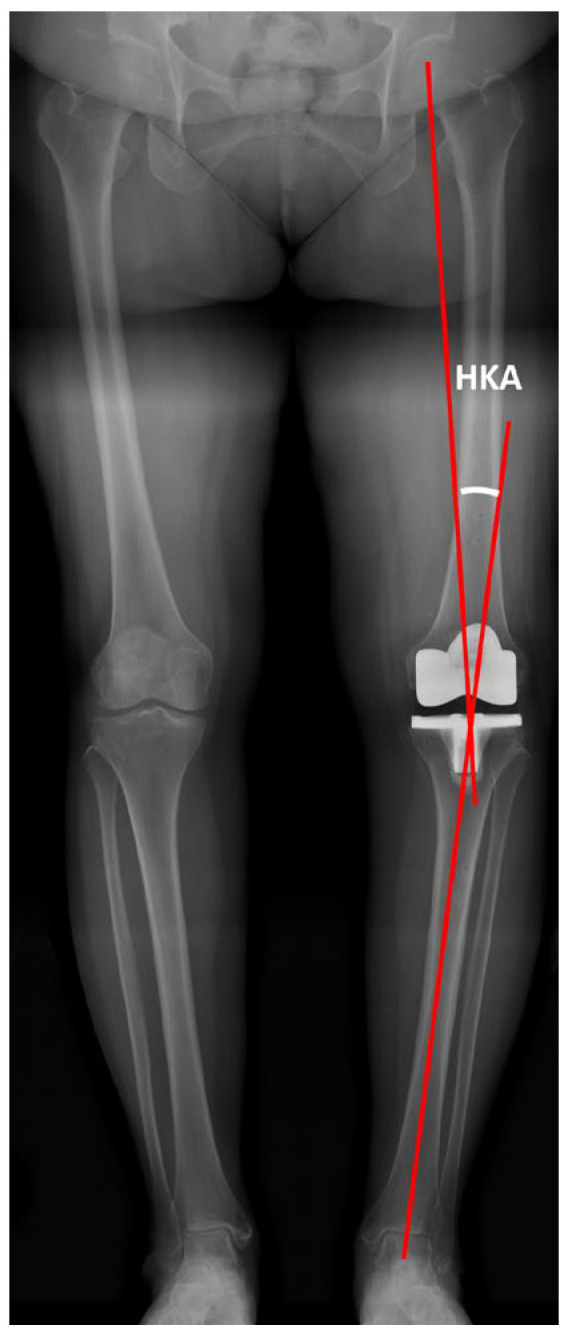
Measurement of the hip–knee–ankle (HKA) angle on full-length standing anteroposterior radiograph.

**Figure 2:**
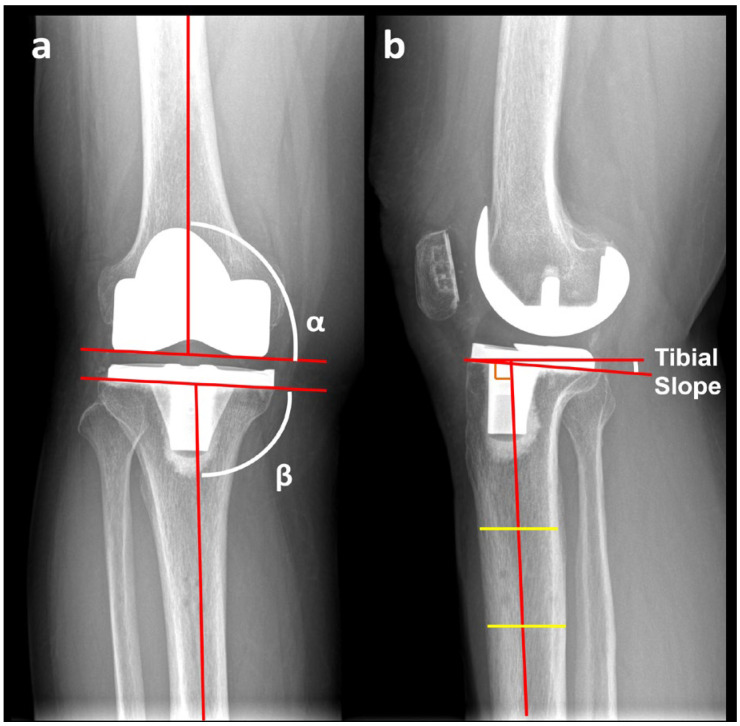
Radiographic measurements of component alignment following total knee arthroplasty. (**a**) Coronal plane: α = femoral component angle, and β = tibial component angle. (**b**) Sagittal plane: tibial slope angle measured between the tibial baseplate and the line perpendicular to the tibial anatomic axis (two-point method).

Standardized standing long-leg anteroposterior and lateral radiographs were obtained preoperatively (closest available to the surgery date) and at 6–12 weeks postoperatively as part of routine follow-up. The HKA angle was measured as the angle between the mechanical axes of the femur and tibia (positive values indicating varus) (Figure 1) [20]. Coronal component alignment included the femoral α angle (angle between a line parallel to the distal femoral condyles and the femoral mechanical axis) and the tibial β angle (angle between the tibial tray surface and the tibial mechanical axis defined from the talar center to the tray midpoint) (Figure 2a) [21]. Posterior tibial slope was measured on standing lateral radiographs as the angle between the tibial baseplate and a line perpendicular to the proximal tibial anatomic axis (two-point method) [22] (Figure 2b). These radiographic parameters were measured by a joint reconstruction fellowship-trained surgeon (Y.-T.L.) and confirmed by a second independent assessor (W.-C.W.).

An HKA within 3 degrees valgus and 6 degrees varus (−3° ≤ HKA < 6°) was considered acceptable [23,24]. A posterior tibial slope between 2° and 8° was also considered acceptable, with values outside these ranges classified as outliers.

### 2.2. Statistical Analysis

Given the observational nature of this study, a post hoc power analysis was performed to estimate the achieved statistical power based on the observed between-group difference in the primary outcome and the final sample size. Using a two-sided α of 0.05, the achieved power was calculated accordingly. Our sample size of 36 patients per group provided a statistical power of 96.6% to detect a difference of 5 points in OKS and >99% to detect a difference of 16 points in KOOS-JR (two-tailed, α = 0.05).

Continuous variables were compared with the independent samples *t*-test. Within-group changes in functional outcomes were evaluated using paired-samples *t*-tests. Categorical variables, including the proportion of patients with a radiographic outlier and those achieving the MCID, were compared using the chi-squared test or Fisher’s exact test, as appropriate (i.e., when any expected cell count was ≤5). All analyses were performed with IBM SPSS Statistics for Windows, version 22.0 (IBM Corp., Armonk, NY, USA). A two-sided *p* < 0.05 was considered statistically significant.

## 3. Results

A total of 72 patients were included in the final analysis after propensity score matching (36 RA-TKAs and 36 M-TKAs). The mean ages of patients receiving RA-TKA and M-TKA were 68.9 and 68.1 years, respectively. The mean BMI was 27.2 in the RA-TKA group and 27.9 in the M-TKA group. The mean operation time was approximately twelve minutes longer in patients receiving RA-TKA (118 min ± 22.8) compared to those receiving M-TKA (106 min ± 26.7) (Table 1).

### 3.1. Functional Outcomes

Both the RA-TKA and M-TKA groups demonstrated significant improvements in functional outcomes postoperatively (Table 2). In the RA-TKA group, the mean Oxford Knee Score (OKS) increased from 25.6 ± 5.4 to 43.9 ± 3.2 (*p* < 0.001), while in the M-TKA group, it improved from 25.5 ± 5.5 to 43.9 ± 2.7 (*p* < 0.001). The KOOS-JR score similarly improved from 61.2 ± 8.6 to 88.9 ± 7.1 in the RA-TKA group (*p* < 0.001) and from 63.3 ± 9.4 to 91.2 ± 8 in the M-TKA group (*p* < 0.001), indicating statistically significant improvements in both cohorts following surgery.

No significant between-group differences were found in preoperative scores for both functional scales (Table 3). When comparing the magnitude of improvement between groups, no statistically significant differences were observed. The mean improvement in OKS was 18.3 ± 5 in the RA-TKA group and 18.4 ± 6 in the M-TKA group (*p* = 0.90). For KOOS-JR, the mean improvement was 27.7 ± 9.2 in the RA-TKA group and 27.9 ± 11 in the M-TKA group (*p* = 0.95).

Regarding achievement of the MCID, all patients in both groups met the MCID threshold for OKS. For KOOS-JR, the proportion of patients reaching the MCID threshold was slightly higher in the RA-TKA group than in the M-TKA group (91.7% vs. 75%), but the difference did not reach statistical significance (*p* = 0.06).

### 3.2. Radiographic Outcomes

No significant differences were observed between the RA-TKA and M-TKA groups in preoperative or postoperative HKA, femoral component α-angle, or tibial component β-angle (Table 4). The mean preoperative HKA was 8.6° ± 3.8 in the RA-TKA group and 8.1° ± 5.4 in the M-TKA group (*p* = 0.76), while the postoperative HKA was corrected to 2.9° ± 3 and 3.3° ± 2.8, respectively (*p* = 0.55). Both femoral (α) and tibial (β) component alignment angles were comparable between groups (*p* = 0.68 and *p* = 0.98, respectively).

In contrast, a significant difference was noted in the posterior tibial slope. The RA-TKA group had a significantly lower mean slope than the M-TKA group (4.3° ± 1.8 vs. 5.9° ± 3.1, *p* = 0.01). Regarding the proportion of radiographic outliers, no difference was found in coronal plane alignment, with 16.7% of RA-TKA and 19.4% of M-TKA cases classified as HKA outliers (*p* = 0.56). However, the RA-TKA group had a significantly lower rate of posterior tibial slope outliers compared with the M-TKA group (16.7% vs. 41.7%, *p* = 0.02).

## 4. Discussion

In this comparative study, RA-TKA achieved 1-year functional outcomes comparable to manual TKA and demonstrated fewer posterior tibial slope outliers. To our knowledge, this is the first study to evaluate the functional and radiological effect of RA-TKA with the ROSA system using a specific medial-congruent (MC) implant.

The adoption of RA-TKA has increased considerably in recent decades. Nevertheless, controversy persists regarding whether RA-TKA yields superior function compared with manual techniques [15,16,25,26]. Most studies report no significant between-group differences in average functional scores; however, some subgroup analyses suggest that advantages for RA-TKA may emerge beyond 6 months [14]. In a meta-analysis that included seven randomized controlled trials, Alrajeb et al. found no significant differences in postoperative Western Ontario and McMaster University score (WOMAC) and Hospital of Special Surgery score (HSS) [15]. Heterogeneity in follow-up intervals and robotic platforms across studies may partly account for inconsistent findings.

Using the same robotic-arm platform (ROSA) as in the present study, Parratte et al. and Kenanidis et al. reported 1-year functional outcomes comparable to manual TKA, with RA-TKA demonstrating superior early results at 6 months [27,28]. Similarly, Khan et al. observed greater short-term (4–6 weeks) KOOS-JR improvements with RA-TKA but no between-group difference at 1 year [29]. Consistent with these reports, RA-TKA and M-TKA in our study showed substantial postoperative gains, although mean 1-year functional scores were similar. Beyond mean changes in functional scores, we assessed whether improvements were clinically meaningful by assessing the 1-year achievement rate of the MCID. Using this patient-centered metric, a greater proportion of patients in the RA-TKA group met the KOOS-JR MCID, but the difference did not reach statistical significance.

Radiographically, the RA-TKA group exhibited fewer posterior tibial slope outliers than the M-TKA group. Prior ROSA-based studies have reported improved alignment accuracy, particularly in the coronal plane [30,31,32]. In the present study, we found that both techniques can reliably meet contemporary coronal targets, including postoperative hip–knee–angle (HKA), femoral component alpha angle, and tibial component beta angle, whereas RA-TKA is better in controlling posterior tibial slope. Tibial slope is clinically relevant, which may influence the flexion gap, cruciate tension in cruciate-retaining (CR) designs, cam–post engagement in posterior-stabilized (PS) designs, mid-flexion stability, and contact mechanics [33,34,35]. Excess or variance in slope can contribute to flexion instability or altered kinematics [36]. In a recent study, Cho et al. reported that restoration of posterior tibial slope after cruciate-retaining TKA (CR-TKA) leads to better clinical outcomes, highlighting the clinical relevance of tibial slope and function after operation [37].

From a technical standpoint, manual tibial slope cuts performed with an extramedullary guide, often without full exposure of the posterior plateau, depend on surface anatomical landmarks (e.g., tibial crest or fibular axis) and are vulnerable to error, particularly in cases with anterior tibial bowing [38]. In contrast, robotic workflows that integrate preoperative planning and intraoperative verification, with multiple registration points on the tibial plateau and at the ankle malleoli, are likely to reduce execution error in the sagittal plane, which is inherently more difficult to standardize manually. Consistent with this, Bollars et al. reported improved accuracy of sagittal alignment and component positioning with RA-TKA compared with manual techniques [39]. A meta-analysis conducted by Riantho et al. found that RA-TKA reduces tibial sagittal component outliers, with an odds ratio of 0.25, an effect that appears more pronounced than for coronal parameters [40]. This highlights the particular advantage of robotics for sagittal accuracy. The medial pivot mechanism makes the MC construct more sensitive to medial flexion gap tension. Ueyama et al. revealed that medial flexion laxity observed using axial radiograph was related to postoperative subjective instability and suboptimal functional outcome [9]. Consistent posterior tibial slope likely harmonizes flexion gap and soft tissue tension, potentially reducing flexion instability. Therefore, precise tibial slope execution is particularly consequential in this implant design and may amplify the benefit of robotic control in the sagittal plane. Nevertheless, whether improved tibial slope consistency translates into better functional outcomes remains uncertain from a statistical standpoint.

The results of the present study support the view that M-TKA remains highly effective, achieving robust functional recovery and reliable coronal alignment. Robotics may add value by improving control of technically demanding parameters, such as tibial slope.

There were several limitations in the present study. First, this was a single-center, retrospective observational analysis with a modest sample size, which may limit statistical power and the precision of effect estimates. Second, the observational study design meant that treatment allocation was not randomized and was determined through surgeon–patient shared decision-making; therefore, selection bias and residual confounding cannot be fully excluded, and the findings should be interpreted as associations rather than causal effects. Furthermore, follow-up was limited to 12 months, which is adequate for early patient-reported outcomes but insufficient to evaluate longer-term endpoints such as implant survivorship, polyethylene wear, progressive or late instability, and late complications. In addition, radiographic differences were primarily observed in posterior tibial slope outliers, while coronal alignment was similar between groups; therefore, any potential advantage of RA-TKA should be framed as improved control of a specific sagittal parameter rather than broadly superior radiographic or functional outcomes. Finally, because all procedures were done using a single robotic platform and a specific MC implant within a standardized pathway, external generalizability to other institutions, robotic systems, implant designs, and alignment philosophies may be limited and should be made with caution.

Further work should examine whether improved tibial slope control translates into durable functional benefit, fewer instability events, and lower revision rates, and should identify the patient groups most likely to benefit. Prospective multicenter studies with larger cohorts, longer follow-up, and ideally randomized designs are warranted to validate these results.

## 5. Conclusions

RA-TKA with an MC bearing provided functional outcomes comparable to M-TKA at 1 year. While mean functional scores and coronal alignment were similar between groups, RA-TKA using the ROSA system was associated with more consistent control of posterior tibial slope and a lower proportion of slope outliers. Longer-term follow-up and randomized trials are warranted to determine whether these advantages translate into sustained functional gains or improved implant survivorship.

## Figures and Tables

**Table 1:** Demographic data and surgical outcome.

Characteristics	Robotic TKA	Manual TKA	*p* Value
N	36	36	
Age	68.9 ± 6.9	68.1 ± 6.3	0.60
Gender †			
Female	22	20	0.63
Male	14	16	
Height (cm)	160.2 ± 8.1	159.8 ± 8.5	0.84
Weight (kg)	69.9 ± 12.3	71.7 ± 12.1	0.54
Body mass index (BMI)	27.2 ± 3.7	27.9 ± 3.8	0.36
Hospital stay (day)	5.2 ± 1.1	5.1 ± 1.6	0.86
Operation time (minute)	118 ± 22.8	106 ± 26.7	0.04 *
Linear thickness (mm)	10.8 ± 0.8	10.8 ± 1	0.70

Student’s *t*-test. * *p* < 0.05; † Chi-squared test.

**Table 2:** Functional outcome improvement after operation.

	Oxford Knee Score	KOOS-JR
ROSA TKR (*n* = 36)		
Preoperative	25.6 ± 5.4	61.2 ± 8.6
Postoperative	43.9 ± 3.2	88.9 ± 7.1
*p* value	0.000 **	0.000 **
Manual TKR (*n* = 36)		
Preoperative	25.5 ± 5.5	63.3 ± 9.4
Postoperative	43.9 ± 2.7	91.2 ± 8
*p* value	<0.01 **	<0.01 **

Paired sample *t*-test. ** *p* < 0.01.

**Table 3:** Functional outcome and status of MCID achievement.

	Robotic TKA (*n* = 36)	Manual TKA (*n* = 39)	*p* Value
Oxford Knee Score (OKS)			
Preoperative	25.6 ± 5.4	25.5 ± 5.5	0.91
Postoperative	43.9 ± 3.2	43.9 ± 2.7	0.97
Improvement	18.3 ± 5	18.4 ± 6	0.90
KOOS-JR			
Preoperative	61.2 ± 8.6	63.3 ± 9.4	0.31
Postoperative	88.9 ± 7.1	91.2 ± 8	0.19
Improvement	27.7 ± 9.2	27.9 ± 11	0.95
Achieve MCID (OKS) ‡			
Yes, *n* (%)	36 (100%)	36 (100%)	
No, *n* (%)	0 (0%)	0 (0%)	
Achieve MCID (KOOS-JR) †			
Yes, *n* (%)	33 (91.7%)	27 (75%)	0.06
No, *n* (%)	3 (8.3%)	9 (25%)	

Student’s *t*-test. ‡ Fisher’s exact test; † Chi-squared test.

**Table 4:** Radiographic outcomes.

	Robotic TKA (*n* = 36)	Manual TKA (*n* = 39)	*p* Value
Femoral component α-angle	89.3° ± 2	89.1° ± 2.2	0.68
Tibial component β-angle	88° ± 2	88° ± 1.7	0.98
Pre-op HKA	8.6° ± 3.8	8.1° ± 5.4	0.76
Post-op HKA	2.9° ± 3	3.3°± 2.8	0.55
HKA outlier † (HKA ≥ 6° or HKA < −3°)	6/36 (16.7%)	7/36 (19.4%)	0.56
Posterior tibial slope	4.3° ± 1.8	5.9° ± 3.1	0.01 *
Tibial slope outlier † (Slope ≥ 8° or <2°)	6/36 (16.7%)	15/36 (41.7%)	0.02 *

Student’s *t*-test. * *p* < 0.05; HKA, Hip-Knee Angle; † Chi-squared test.

## Data Availability

The authors are able to provide the data that supports the findings of this study upon request. Please contact the corresponding authors for further information.

## Abbreviations

The following abbreviations are used in this manuscript:

TKA	Total knee arthroplasty
RA-TKA	Robotic-assisted total knee arthroplasty
M-TKA	Manual total knee arthroplasty
MC	Medial congruent
MP	Medial pivot
OKS	Oxford Knee Score
MCID	Minimal clinically important difference
rKA	Restricted kinematic alignment
HKA	Hip–knee angle
LDFA	Lateral distal femoral angle
MPTA	Medial proximal tibial angle
